# Phenotypic and genotypic characterization of biofilm producing clinical coagulase negative staphylococci from Nepal and their antibiotic susceptibility pattern

**DOI:** 10.1186/s12941-021-00447-6

**Published:** 2021-05-31

**Authors:** Sarita Manandhar, Anjana Singh, Ajit Varma, Shanti Pandey, Neeraj Shrivastava

**Affiliations:** 1 Tri-Chandra Multiple College, Tribhuvan University, Kathmandu, Nepal; 2 Amity Institute of Microbial Technology, Amity University Uttar Pradesh, Noida, UP 201303 India; 3 Central Department of Microbiology, Tribhuvan University, Kathmandu, Nepal; 4 The University of Southern Mississippi, Hattiesburg, MS 39406 USA

**Keywords:** Coagulase-negative staphylococci, Biofilm production, Antibiotic susceptibility

## Abstract

**Background:**

Coagulase-negative staphylococci (CNS) survive as commensals of skin, anterior nares and external canals of human and were regarded as non-infectious pathogens. However, they are emerging as a major cause of nosocomial infectious due to their ability to form biofilms and high resistance to several classes of antibiotics. This study examines the biofilm forming abilities of 214 clinical CNS isolates using phenotypic and genotypic methods, and determines their antibiotic susceptibility patterns.

**Methods:**

A total of 214 clinical isolates collected from different clinical samples were identified as CNS and their antibiotic susceptibility determined by CLSI guidelines. The biofilm forming ability of all isolates was determined by three phenotypic methods; Congo red agar (CRA) method, tube adherence method (TM) and tissue culture plate (TCP) method and by genotypic method for the detection of *icaAD* genes.

**Results:**

Among all the isolates, *S. epidermidis* (57.5%) was found the most frequently, followed by *S. saprophyticus *(18.7%)*, S. haemolyticus *(11.2%)*, S. hominis* (7%), and *S. capitis* (5.6%). Antibiotic susceptibility pattern demonstrated 91.6% isolates were resistant to penicillin and 66.8% to cefoxitin while 91.1% isolates were susceptible to chloramphenicol. Constitutive and inducible clindamycin resistant phenotype as measured by D-test was seen among 28% and 14.5% of isolates respectively. Tissue culture plate method detected biofilm production in 42.1% isolate followed by 31.8% through tube method while 20.1% isolates were found to produce slime in Congo red agar method. The genotypic assay revealed presence of *icaA* and *icaD* genes in 19.2% isolates.

**Conclusion:**

The study shows a high prevalence of biofilm formation and inducible clindamycin resistance in CNS isolates, indicating the importance of *in-vitro* biofilm production test and D-test in routine laboratory diagnostics. Implementation of efficient diagnostic techniques for detection of biofilm production in clinical samples can help manage staphylococcal infections and minimize risks of treatment failures in hospitals.

## Background

Presently, the wide-spread use of implants in modern healthcare facilities has substantially increased the risk of device associated infections, which in turn has drastically increased the rate of mortality and morbidity. Non-pathogenic indigenous microbiota of skin, nares and other mucosal surfaces of human, coagulase-negative staphylococci (CNS) are opportunistic pathogens accounting as one of the most common etiologic agents of device related infections [1, 2]. Over the past few decades, CNS, especially *Staphylococcus epidermidis* has emerged as a major cause of nosocomial infections [3–5]. The pathogen, now regarded as a life threatening, causes septicemia, meningitis, endocarditis and other serious invasive infections. Immunocompromised individuals or patients undergoing treatment with indwelling devices such as catheters and tips are more vulnerable to contract the CNS infections. Therefore, paradoxically, medical devices aimed to improve the treatment outcome, have often contributed to the prevalence of nosocomial infections [1, 5, 6], increasing the clinical significance of CNS.

Biofilm production is one of the major arsenals of saprophytic microbiota to become an opportunistic pathogen [7, 8]. Through biofilms, the CNS can adhere to and colonize on biotic as well as abiotic surfaces. Likewise, the damaged host tissue caused by prolonged antibiotic use and the implanted devices facilitate the survival, proliferation, and virulence of these pathogens [4, 6]. The biofilm of CNS is composed of the layer of extracellular polymeric substance called polysaccharide intercellular adhesion (PIA) matrix, which is encoded by *ica* operon (*icaADBC* genes) [9]. Beneath the biofilm matrix, bacteria are protected from physical, chemical, and biological stresses imposed by the antibiotics and the host immune cells [4]. Indeed, increased evidences show that the bacteria embedded within biofilms are difficult to kill by the antibiotics that are effective against planktonic cells, leading to the treatment failures of biofilm infections [1, 10, 11]. Moreover, the dispersal phenomenon of biofilms also serves as a source to metastatic infections [1, 4, 10]. There are mounting evidences of antibiotic resistance among biofilm forming clinical staphylococcal isolates. Furthermore, polymicrobial proximity within the biofilm might facilitate horizontal exchange of genetic information leading to development of antibiotic resistant clonal population [7]. Therefore, considering the clinical significance of biofilm associated infections, prevention and management of CNS infections remain a priority for the betterment of public health. This warrants the implementation of efficient methods to detect biofilm production among clinical samples in routine laboratory diagnosis.

The antimicrobial susceptibility testing (AST) of a clinical isolate is crucial for the optimal antimicrobial treatment of infected patients. This practice has been even more critical considering the single or multidrug resistant microorganisms [12]. Studies have reported clinical antibiotic resistance including that against erythromycin, macrolides, lincosamides and streptogramins type B (MLS_B_) in clinical CNS isolates [13, 14]. Many studies have reported the prevalence of antibiotic resistance including MLS_B_ resistance *S. aureus* isolates from different regions of Nepal [15–22]. Recent studies although have reported antibiotic resistance and biofilm in clinical CNS isolates [22, 23], the prevalence of MLS_B_ resistance in the CNS isolates are lacking in Nepal. Herein, we report the prevalence of antibiotic resistance and biofilm production among clinical CNS isolates collected from two tertiary care hospitals of Nepal.

## Methods

### Isolation and identification of coagulase-negative staphylococci (CNS)

A descriptive cross sectional study was carried out at two tertiary care hospitals of Nepal; B & B hospital and Kathmandu Institute of Technology (KIST) Medical College & Hospital. A total of 214 isolates were collected from various clinical samples including central venous catheter (CVC), catheter tip, suction tip, drain tip, Double J (DJ) stent, tracheal tip, endotracheal tip, blood, wound/pus and urine.

The elution of the bacterial cells from CVC or catheters was done by following standard microbiological protocol routinely followed in the hospital. The catheter tips were collected in sterile container and then mixed with 2 ml of nutrient broth (NB). After mixing by vortexing, loop-full of the suspension was streaked on MacConkey Agar (MA), Blood Agar (BA) plate and further processed for bacterial identification. The isolates were identified as staphylococci according to the standard microbiological procedure which included colony morphology on BA and mannitol salt agar (MSA), Gram staining and biochemical tests including catalase, slide and tube coagulase tests and oxidative/fermentative (O/F) test [25]. Once the isolates were identified as CNS, they were classified into species following simplified scheme proposed by Cunha et al., [26] using several biochemical tests. After identification of species, the isolates were stored in tryptic soy broth (TSB) with glycerol in freezer at −20 °C for further use.

### Phenotypic and genotypic characterization of biofilm formation

Both phenotypic and genotypic methods were used for the detection of biofilm production in all the isolates. ATCC 35983 *S. epidermidis* strain was used as positive control for biofilm production in all assays performed.

### Congo red agar method (CRA)

The screening of biofilm production was performed using CRA media; a qualitative method as described by Freeman et al., [27]. The medium composed of Brain Heart Infusion (BHI) agar supplemented with sucrose and a dye; Congo red. These plates were inoculated with CNS isolates, incubated aerobically at 37 ºC for 24 h and were interpreted based on the qualitative observation of colored colonies formation on the CRA plates. The black colonies with dark consistency were regarded as strong biofilm producers while the pink colonies were regarded as biofilm non-producers. The experiments were performed in triplicates and repeated three times.

### Tube adherence method (TM)

This qualitative method for the detection of biofilm formation was performed as described by Christensen et al. [28]. A loop-full of microorganism was inoculated in trypticase soy broth (TSB) supplemented with 1% glucose. The tubes were incubated at 37 ºC for 24 h. The tubes were decanted, washed with PBS (pH 7.3) for 4 times and dried. Tubes were then stained with 0.1% crystal violet for 15 min. Excess stain was removed by washing with deionized water for 3 times. The tubes were then dried in inverted position and observed for biofilm formation. In this assay, biofilm formation was considered positive when a visible film was observed along the inner wall and bottom of tube. Depending on this, isolates were scored as 0 for absence, + for weak, +  + for moderate, and +  +  + for strong biofilm formation. The experiments were performed in triplicates and repeated three times.

### Tissue culture plate (TCP) method

All the isolates were screened for their ability to produce biofilm by this quantitative method as previously described by Christensen et al. [28] with slight modification [22]. In this assay, a loop-full of organism was inoculated in 5 mL TSB supplemented with 1% glucose and incubated at 37º C for 24 h. The overnight culture was diluted 1:100 with fresh media and 0.2 mL of this diluted culture was inoculated into individual wells of sterile polystyrene 96 well flat bottom tissue culture plates and incubated at 37 ºC for 24 h. After incubation, the content of tissue culture plate was removed by gentle tapping, and washed with PBS (pH 7.2) 4 times to remove free flowing planktonic bacteria. Biofilms formed by adherent sessile bacteria in the plate were fixed with 2% sodium acetate. It was then stained with 0.1% crystal violet for 15 min at room temperature. Excess stain was rinsed off by washing with deionized water for 4 times and plates were dried. Optical density (OD) of stained adherent bacteria was measured with micro ELISA auto reader at OD 630_ nm_. OD values from sterile medium, fixative and dye were averaged and subtracted from all test values. The experiments were performed in triplicates and repeated three times. Bacterial adherence was classified based on OD values of the individual isolates. Mean OD value < 0.120, 0.120–0.240 and > 0.240 were classified as non/weak, moderate and strong biofilm adherence respectively.

### Detection of ica genes

The genomic DNA was extracted as previously described [22] using the DNA extraction Kit (Thermo Fischer). The sequences of *icaA* and *icaD* (accession number U43366) were taken from the GenBank sequence of the National Center for Biotechnology Information (NCBI) database. Primers specific for *icaA* and *icaD* were forward 5′-TCTCTTGCAGGAGCAATCAA, reverse 5′-TCAGGCACTAACATCCAGCA generating a product size of 188 bp and forward 5′-ATGGTCAAGCCCAGACAGAG, reverse 5′-CGTGTTTTCAACATTTAATGCAA with a product size of 198 bp respectively [22]. A 25 μl of reaction mixture consisted of MgCl_2_ (2.5 mM), Taq DNA polymerase (1U), each dNTPs (100 μM), each primer (1 μM) and DNA extract (200 ng). DNA amplification was carried out with following parameters: preheating at 95 °C for 5 min followed by 35 cycles of amplification (denaturation at 94 °C for 30 s, annealing at 55 °C for 30 s and elongation at 72 °C for 30 s) and termination at 72 °C for 2 min. The PCR product was analyzed in 2% agarose gel stained with SYBR safe dye (Invitrogen).

### Antimicrobial susceptibility testing (AST)

AST of the isolates was performed on Mueller Hinton Agar (MHA) by modified Kirby-Bauer disk diffusion method recommended by clinical laboratory standard institution (CLSI) guidelines [29]. The antimicrobial discs (HiMedia Laboratories) used in the study were: penicillin (10 units), ciprofloxacin (30 µg/disc), tetracycline (30 µg/disc), clindamycin (2 µg/disc), chloramphenicol (30 µg/disc), cefoxitin (30 µg/disc), erythromycin (15 µg/disc), cotrimoxazole (1.25/23.75 µg/disc) and gentamicin (10 µg/disc). The cefoxitin disc was used to detect methicillin resistance. The *Staphylococcus aureus* ATCC 25923 was used as reference strain for analyzing AST results.

### Screening of inducible clindamycin resistance

The double disc diffusion test or D zone test outlined in CLSI document M100-S25 [29] was performed to determine if the erythromycin resistant isolates expressed inducible clindamycin resistance. Erythromycin (15 µg) disc was placed at a distance of 15 mm (edge to edge) from clindamycin (2 µg) on Mueller Hinton agar plates previously inoculated with 0.5 McFarland bacterial suspensions. Plates were analyzed after 18 h of incubation at 37 °C. Interpretation of the inhibition zone diameters was as follows: If an isolate was erythromycin resistant and clindamycin susceptible, with a D-shaped inhibition zone around the clindamycin disc, it was considered positive for inducible resistance (D-test positive, iMLS_B_ phenotype). If the isolate was erythromycin resistant and clindamycin susceptible, with both zones of inhibition showing a circular shape, the isolate was considered to be negative for inducible resistance (D test negative, MS phenotype), but to have an active efflux pump. If the isolate was resistant to both drugs, it was considered to have the macrolide–lincosamide–Streptogramin B constitutive (cMLS_B_ phenotype) [30].

### Statistical analysis

The statistical analysis was performed using SPSS 17.0 (SPSS Inc., Chicago, USA) software. Chi-square test was used to compare between groups and *P* values < 0.05 were considered statistically significant.

## Results

### Isolation and identification of CNS

**Fig. 1:**
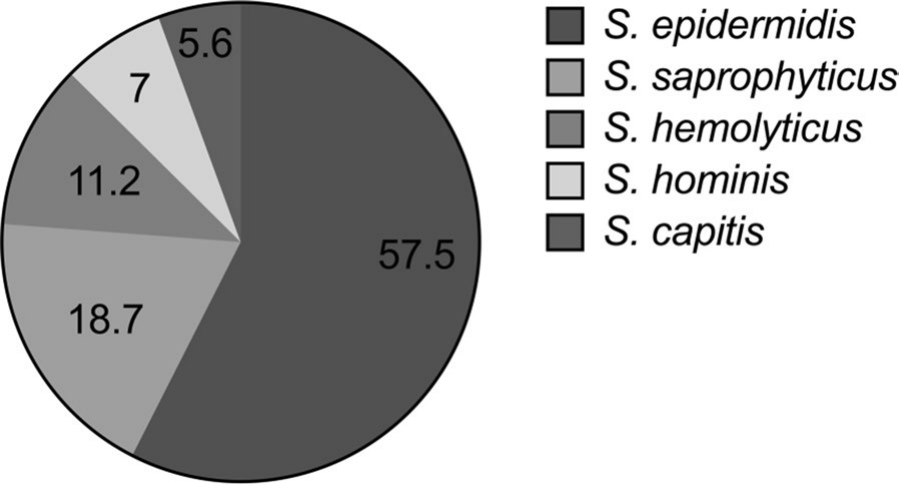
Percentage of CNS isolated from various clinical samples

A total of 214 isolates were identified as CNS from various clinical samples following standard microbiological procedure using different biochemical tests [25]. Five species were identified among all CNS isolates including *S. epidermidis* (57.5%); the most frequently isolated species followed by *S. saprophyticus (*18.7%)*, S. haemolyticus (*11.2%)*, S. homonis* (7%) and *S. captis* (5.6%) (Fig. 1).

### Frequency of CNS isolated from different clinical specimens

*S. epidermidis* was isolated from all specimen types received in the laboratory. It was most frequently isolated from blood (52, 42.3%) followed by wound/pus (34, 27.6%). Similarly, *S. saprophyticus* was also mostly isolated from blood (12, 30%) and wound/pus (12, 30%). Different implant devices were also found to harbor CNS. All CNS were isolated from catheter tip except *S. hominis.* However*, S. hominis* were isolated from CVC, blood, wound/pus and urine. On the other hand, *S. saprophyticus* and *S. haemolyticus* were not isolated from suction tip, drain tip, tracheal tip and endotracheal tip. Similarly, *S. capitis* were isolated only from CVC, catheter tip, blood, and wound/pus (Table 1)*.*

**Table 1:** Frequency of CNS isolated from different clinical specimens

CNS	Clinical specimen							Total
	CVC	Catheter tip	Blood	Wound/pus		Urine	Others	
*S. epidermidis*	8 (6.5%)	14 (11.4%)	52 (42.3%)		34 (27.6%)	11(8.9%)	4 (3.3%)	123 (57.5%)
*S. saprophyticus*	2 (5.0%)	2 (5.0%)	12 (30.0%)		12 (30.0%)	11(27.5%)	1(2.5%)	40 (18.7%)
*S. hemolyticus*	6 (25.0%)	6 (25.0%)	5 (20.8%)		5 (20.8%)	1(4.2%)	1(4.2%)	24 (11.2%)
*S. hominis*	1 (6.7%)	–	10 (66.7%)		3 (20.0%)	1 (6.7%)	–	15 (7.0%)
*S. capitis*	3 (25.0%)	3 (25.0%)	3 (25.0%)		2 (16.7%)	–	1(8.3%)	12 (5.6%)

*CVC* central venous catheter; others (Suction tip, Drain tip, Double J stent, Tracheal tip, Endotracheal tube)

### Antibiotic susceptibility pattern among CNS isolates

Antibiotic susceptibility testing of 9 clinically relevant antibiotics was performed against all the collected isolates. These antibiotics were selected because of their common use in clinical practice in hospitals of Nepal. Among all CNS, 91.6% isolates were found to be resistant to penicillin. Mostly, *S. epidermidis* (114, 92.7%), *S. saprophyticus* (35, 87.5%) and *S. haemolyticus* (23, 95.8%) were susceptible to chloramphenicol. Similarly, *S. capitis* (12, 100%) and *S. hominis* (14, 93.3%) were susceptible to tetracycline (Table 2). Cefoxitin disc was used to detect methicillin resistance, which was observed in 66.8% CNS isolates. All five CNS species were found to be methicillin resistant with 73 (59.3%) *S. epidermidis,* 29 (72.5%) *S. saprophyticus,* 21 (87.5%) *S. haemolyticus,* 10 (66.7%) *S. hominis* and 10 (83.3%) *S. capitis* (Table 2).

**Table 2:** Antimicrobial susceptibility pattern of CNS isolates

Antibiotics	Total (n = 214)		*S. epidermidis* (n = 123)		*S. saprophyticus* (n = 40)		*S. hemolyticus* (n = 24)		*S. hominis* (n = 15)		*S. capitis* (n = 12)	
	R	S	R	S	R	S	R	S	R	S	R	S
CNS												
Penicillin	196 (91.6%)	18 (8.4%)	111 (90.2%)	12 (9.8%)	39 (97.5%)	1 (2.5%)	23 (95.8%)	1 (4.2%)	12 (80%)	3 (20%)	11 (91.7%)	1 (8.3%)
Ciprofloxacin	76 (35.5%)	138 (64.5%)	39 (31.7%)	84 (68.35)	7 (17.5%)	33 (82.5%)	17 (70.8%)	7 (29.2%)	5 (33.3%)	10 (66.75)	8 (66.7%)	4 (33.3%)
Tetracycline	27 (12.6%)	187 (87.4%)	11 (8.9%)	112 (91.1%)	8 (20%)	32 (80%)	7 (29.2%)	17 (70.8%)	1 (6.7%)	14 (93.3%)	-	12 (100%)
Clindamycin	63 (29.4%)	151 (70.6%)	30 (24.4%)	93 (75.6%)	13 (32.5%)	27 (67.5%)	9 (37.5%)	15 (62.5%)	5 (33.3%)	10 (66.7%)	6 (50%)	6 (50%)
Chloramphenicol	19 (8.9%)	195 (91.1%)	9 (7.3%)	114 (92.7%)	5 (12.5%)	35 (87.5%)	1 (4.2%)	23 (95.8%)	2 (13.3%)	13 (86.7%)	2 (16.7%)	10 (83.3%)
Cefoxitin	143 (6.5%)	71 (33.2%)	73 (59.3%)	50 (40.7%)	29 (72.5%)	11 (27.5%)	21 (87.5%)	3 (12.5%)	10 (66.7%)	5 (33.3%)	10 (83.3%)	2 (16.7%)
Erythromycin	155 (72.4%)	59 (27.6%)	80 (65%)	43 (35%)	34 (85%)	6 (15%)	21 (87.5%)	3 (12.5%)	10 (66.7%)	5 (33.3%)	10 (83.3%)	2 (16.7%)
Cotrimoxazole	80 (37.4%)	134 (62.6%)	46 (37.4%)	77 (62.6%)	14 (35%)	26 (65%)	10 (41.7%)	14 (58.3%)	6 (40%)	9 (60%)	4 (33.3%)	8 (66.7%)
Gentamicin	45 (21%)	169 (79%)	17 (13.8%)	106 (86.2%)	8 (20%)	32 (80%)	14 (58.3%)	10 (41.7%)	–	15 (100%)	6 (50%)	6 (50%)

*R* Resistant, *S* Sensitive

### Inducible clindamycin resistance

Clindamycin is a useful drug in the treatment of serious infections caused by Staphylococci due to its excellent tissue penetration, good oral absorption, and is an alternative to penicillin allergic patients. However, it has been indicated that approximately 45% of erythromycin resistant *S. aureus* isolates have inducible MLS_B_ resistance that would go unrecognized if erythromycin and clindamycin disc are not placed appropriately during routine antibiotic susceptibility test [29, 31]. Among 214 CNS, 155 (72.4%) were resistant to erythromycin. MS phenotype (D test) was performed for these isolates and showed that 60 (28%) isolates were resistant to both erythromycin and clindamycin indicating constitutive MLS_B_ phenotype. Out of 151 clindamycin sensitive isolates, positive D test was observed among 31(14.5%) isolates, indicating inducible MLS_B_ phenotype. Negative D-test results were obtained among 70 (32.7%) isolates, whereas, the remaining 53 (24.8%) isolates were susceptible to both erythromycin and clindamycin. Constitutive and inducible MLS_B_ phenotype was 51 (23.8%) and 25 (11.7%) among methicillin resistant CNS and 9 (4.2%) and 6 (2.8%) in methicillin sensitive CNS respectively. Both constitutive and inducible MLS_B_ phenotype was predominant among MRCNS as compared to MSCNS (Table 3).

**Table 3:** Erythromycin and clindamycin susceptibility testing

Phenotypes	MRCNS		MSCNS		Total	
	(n)	%	(n)	%	(n)	%
E-S, CD-S	18	8.4	35	16.4	53	24.8
E-R, CD-R (constitutive MLS_B_)	51	23.8	9	4.2	60	28
E-R, CD-S (inducible MLS_B_, D-positive)	25	11.7	6	2.8	31	14.5
E-R, CD-S (MS, D-negative)	49	22.9	21	9.8	70	32.7
Total	143	66.8	71	33.2	214	100

*MRCNS* Methicillin resistant CNS, *MSCNS* Methicillin sensitive CNS, *E* Erythromycin, *CD* Clindamycin, *R* Resistant, *S* Sensitive, *MLS*_*B*_ Macrolides, Lincosamides and Streptogramin B

### Biofilm formation among CNS isolates

Biofilm production was assessed by both phenotypic and genotypic methods. The slime production ability of identified clinical CNS isolates was screened by CRA method. Among 214 CNS, 20 (9.3%) isolates produced black colonies indicating strong positive for polysaccharide production. The qualitative assessment of the amount of biofilm production was done by tube adherence method (TM) which showed strong production (+ + +) among 44 (20.6%) isolates and moderate biofilm production among 24 (11.2%) isolates. Biofilm production was determined quantitatively by tissue culture plate (TCP) method that demonstrated strong and moderate biofilm producers in 35 (16.4%) and 55 (25.7%) CNS isolates. Among all CNS species, *S. epidermidis* was the most frequent species to produce biofilm in all phenotypic methods. In addition, genotypic assay also revealed this species harboring both *icaA* and *icaD* genes more frequently. The *ica* genes were detected in 41 (19.2%) of all CNS isolates. Among all methods, TCP detected biofilm production in a greater number of isolates in all species (Table 4).

**Table 4:** Determination of biofilm formation in CNS by genotypic and phenotypic methods

Biofilm	CNS isolates					Total (n = 214)
	*S. epidermidis*	*S. saprophyticus*	*S. haemolyticus*	*S. hominis*	*S. capitis*	
	(n = 123)	(n = 40)	(n = 24)	(n = 15)	(n = 12)	
Congo red agar (CRA)						
Strong	12 (9.8%)	2 (5.0%)	3 (12.5%)	3 (20.0%)	-	20 (9.3%)
Moderate	12 (9.8%)	2 (5.0%)	5 (20.8%)	2 (13.3%)	2 (16.7%)	23 (10.7%)
Weak/none	99 (80.5%)	36 (90.0%)	16 (66.7%)	10 (66.7%)	10 (83.3%)	171 (79.9%)
Tube adherence method (TM)						
Strong	33 (26.8%)	4 (10.0%)	2 (8.33%)	3 (20.0%)	2 (16.7%)	44 (20.6%)
Moderate	9 (7.3%)	6 (15.0%)	6 (25.0%)	3 (20.0%)	-	24 (11.2%)
Weak/none	81 (65.9%)	30 (75.0%)	16 (66.7%)	9 (60.0%)	10 (83.3%)	146 (68.2%)
Tissue culture plate (TCP)						
Strong	23 (18.7%)	8 (20.0%)	3 (12.5%)	1 (6.7%)	-	35 (16.4%)
Moderate	32 (26.0%)	9 (22.5%)	4 (16.7%)	7 (46.7%)	3 (25.0%)	55 (25.7%)
Weak/none	68 (55.3%)	23 (57.5%)	17 (70.8%)	7 (46.7%)	9 (75.0%)	124 (57.9%)
*ica* gene						
Present	29 (23.6%)	5 (12.5%)	2 (8.33%)	3 (20.0%)	2 (16.7%)	41 (19.2%)
Absent	94 (76.4%)	35 (87.5%)	22 (91.7%)	12 (80.0%)	10 (83.3%)	173 (80.8%)

### Determination of biofilm formation among methicillin resistant CNS isolates

The biofilm production as detected by different phenotypic methods were higher in methicillin resistant than methicillin sensitive isolates but was not found to be statistically significant. Similarly, the *ica* genes were also found in higher number among methicillin resistant CNS than methicillin sensitive CNS but it was not found to be statistically significant (Table 5).

**Table 5:** Biofilm formation in CNS in relation to methicillin susceptibility

Biofilm	MRCNS	MSCNS	*p*-value
Congo red agar (CRA)			0.322
Present	26 (18.2%)	17 (23.9%)	
Absent	117 (81.8%)	54 (76.1%)	
Tube adherence method (TM)			0.447
Present	43 (30.1%)	25 (35.2%)	
Absent	100 (69.9%)	46 (64.8%)	
Tissue culture plate (TCP)			0.529
Present	58 (40.6%)	32 (45.1%)	
Absent	85 (59.4%)	39 (54.9%)	
*ica* genes			0.824
Present	28 (19.6%)	13 (18.3%)	
Absent	115 (80.4%)	58 (81.7%)	

*MRCNS* Methicillin resistant CNS, *MSCNS* Methicillin sensitive CNS

### The icaAD genes are present in all clinical sample type

**Fig. 2:**
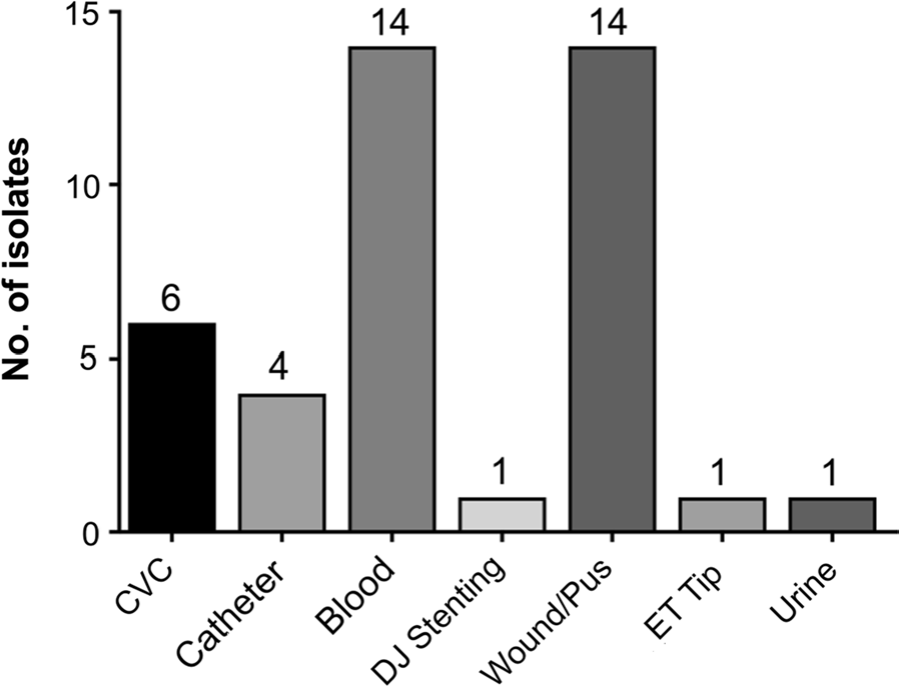
Distribution of CNS isolates possessing *ica* genes in different clinical specimens

**Fig. 3:**
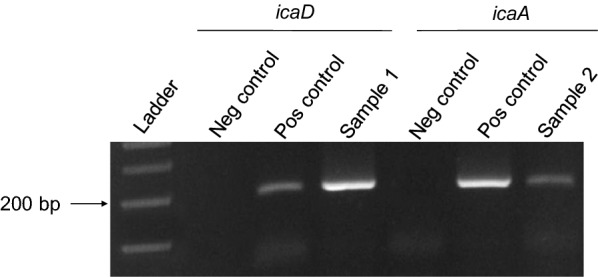
Representative picture of PCR amplification of *icaA* (188 bp) and *icaD* (198 bp) genes in CNS isolates

The study revealed that 14 (17.07%) CNS isolates from blood samples (n = 82) and 14 (25%) from wound/pus samples (n = 56) harbored *ica* genes. Likewise, *ica* genes were also present in six (30%) biomaterial isolates collected from CVC (n = 20), four (16%) from catheters (n = 23), and one each from DJ stenting (n = 2), ET tip (n = 1), and urine samples (n = 24) (Fig. 2). The identification of *icaAD* genes was done by PCR of DNA extracted from the CNS isolates using the primer listed in method section (Fig. 3).

### Antibiotic susceptibility pattern of isolates that harbor ica genes

The antibiotic susceptibility patterns of the isolates harboring *ica* genes were determined. The study showed no significant difference in antibiotic susceptibility between *icaAD* positive and *icaAD* negative isolates. Methicillin resistance was observed more among the isolates harboring *ica* genes than those lacking it. Altogether, the result shows that not only biofilm formation but other factors are responsible for antibiotic resistance among CNS (Table 6).

**Table 6:** Antibiotic susceptibility pattern of *ica* positive isolates

Antibiotics	*ica* genes				*p-*value
	Present		Absent		
	Susceptible	Resistant	Susceptible	Resistant	
Penicillin	4 (1.9%)	37 (17.3%)	14 (6.5%)	159 (74.3%)	0.119
Ciprofloxacin	28 (13.1%)	13 (6.1%)	110 (51.4%)	63 (29.4%)	0.334
Tetracycline	38 (17.8%)	3 (1.4%)	149 (69.6%)	24 (11.2%)	6.590
Clindamycin	32 (15.0%)	9 (4.2%)	119 (55.6%)	54 (25.2%)	3.332
Chloramphenicol	38 (17.8%)	3 (1.4%)	157 (73.4%)	16 (7.5%)	0.231
Cefoxitin	13 (6.1%)	28 (13.1%)	58 (27.1%)	115 (53.7%)	0.049
Erythromycin	15 (7.0%)	26 (12.1%)	44 (20.6%)	129 (60.3%)	2.256
Cotrimoxazole	29 (13.6%)	12 (5.6%)	105 (49.1%)	68 (31.8%)	1.460
Gentamicin	35 (16.4%)	6 (2.8%)	134 (62.6%)	39 (18.2%)	1.464

## Discussion

The coagulase-negative staphylococci (CNS) including *S. epidermidis* are ubiquitous in nature; reside on the skin of healthy individuals as normal flora. In fact, due to this phenomenon, CNS has been emerged as common nosocomial pathogens. In addition, the ability to form biofilms on biotic as well as abiotic surfaces have made them successful human pathogens causing persistent infections leading to serious health problems. Given that biofilm is an important virulence factor that is mostly associated with antibiotic resistance for these pathogens, early detection in clinical specimen would impose significant impact in management of staphylococcal nosocomial infections. Taking this into consideration, the study was carried out to investigate the prevalence of biofilm producing CNS and antibiotic resistant strains in different clinical samples collected from two tertiary care hospitals of Nepal.

Among 214 CNS, *S. epidermidis* was the most common isolate accounting for more than half (57.4%) of total numbers (n = 123*)* followed by *S. saprophyticus* (n = 40, 18.6%), *S. haemolyticus* (n = 24, 11.2%), *S. hominis* (n = 15, 7%) and *S. capitis* (n = 12, 5.6%) (Table 1). Previous studies have also reported *S. epidermidis* as the most common isolates among CNS [24, 32–34]. While *S. epidermidis* was isolated form all clinical samples, it was more commonly isolated from blood, wound/pus. *S. saprophyticus* along with blood and wound/pus was also isolated from urine. The high number of *S. saprophyticus* in urine is consistent with the previous studies [24, 35] may be due to its adhering capacity to wall of urinary tract. Importantly, all CNS species were isolated from different medical devices indicating their ability to cause device associated infections.

Antibiotic resistance is a major human health problem. The present result also demonstrated that majority of isolates were 91.6% and 72.4% resistant to penicillin and erythromycin respectively. However, the study also demonstrated majority of isolates being susceptible to chloramphenicol (91.1%) and tetracycline (87.4%). Such high frequency of susceptibility pattern of chloramphenicol is consistent with the previous study by [24, 33, 36, 37], but their study showed lower susceptibility in case of erythromycin and penicillin. Indeed, similar susceptibility profile for all CNS strains was found as that of *S. epidermidis* strains. This indicates CNS are still susceptible to the first line drug and being cheap, these antibiotics could be used for the treatment of CNS infections caused by CNS in a resource limiting country like Nepal.

Among all CNS isolates, constitutive MLS_B_ were present among 51 (23.8%) isolates and inducible MLS_B_ among 25 (11.7%) isolates. While similar frequency of inducible MLS_B_ was demonstrated by Perez et al., [38] as high as 50% CNS were found to be positive in study by Schreckenberger et al., [14]. These results indicate that constitutive and inducible MLS_B_ resistance is dependent on the hospitals and the geographical area. Nonetheless, chances of inducible MLS_B_ can be reduced by implementing reliable diagnostics of MLS_B_ in the clinical samples.

Biofilm formation remains the most important mechanism of pathogenicity among staphylococci [10]. Although CRA and TM methods detected the biofilm production in a smaller number of isolates, TCP method was able to detect biofilm production in 90 (42.1%) isolates. Similar frequency of biofilm production in clinical CNS isolates was reported previously [39–41]. However, the higher frequency (65.38%) of biofilm production was reported by Shrestha et al., [23] in a study, conducted in tertiary care hospital of eastern Nepal as well as the study by Oliveira et al., [42], who reported 75% of CNS producing biofilm. Likewise, PCR amplification in our study revealed only 41 (19.2%) isolates possessing both *icaA* and *icaD* genes. This rate is lower as compared to the previous studies [33, 40]. The possible reasons are the various factors such as environment, nutrition, sub-inhibitory concentration of certain antibiotics, and stress (temperature, osmolarity) might play a significant role in biofilm formation resulting in varied frequency of biofilm producers among clinical isolates [43–47].

Among 41 *icaAD* positive isolates, only 7 (17%) showed the positive result in CRA method. This is in contrast to the previous studies by Zhou et al., [48] who reported all *icaAD* positive isolates also produced black colonies on CRA method. Similarly, good correspondence between possession of *icaAD* genes and CRA positivity was reported by de Silva et al., [2]. Our result shows that not all biofilm producers as per phenotypic assays possessed *ica* genes. Several previous studies have reported this phenomenon [49–52]. We speculated that while most of the staphylococcal isolates which form biofilm are dependent on the *icaAD* genes, other factors such as teichoic acids also contribute to form biofilm [33]. It may also be possible that inactivation of *ica* operon occurs due to the insertion of the IS256 insertion sequence. Similarly, the *ica*-negative, non-slime-producing isolates likely represent strains with alternate means of adhesion, such as microbial surface components recognizing adhesive matrix molecules (MSCRAMMs). These findings reinforce the opinion that several mechanisms besides slime production are responsible for bacterial adhesion and hence biofilm production [43].

Our study revealed *ica* genes were present mostly among blood and wound/pus samples (14 isolates each). Our results are consistent with the study previously performed [40] where quarter of the isolates from blood cultures and catheter tips produced biofilm. However, in contrast, we detected lesser frequency of biofilm production in the biomaterial sample like CVC, catheter, DJ stenting and ET tip. Interestingly, we did not observe significant difference of antibiotic resistance among biofilm producers and non-producers. Similar results were also reported previously [53] with no difference in antimicrobial resistance between biofilm-producing and non-producing *S. epidermidis*. Nonetheless, altogether, the percentage of biofilm production was higher among *S. epidermidis* than in other CNS isolates indicating that biofilm production is an important virulence factor for the pathogenicity of *S. epidermidis*. However, the results also support the idea that neither biofilm nor the *icaAD* genes could alone be used as biomarker of clinical significance, as suggested previously [2].

## Conclusion

This study identified *S. epidermidis* as the most frequent species (57.5%) with the highest rate of biofilm production in all CNS examined. Among all phenotypic methods, TCP method detected biofilm production in higher percentage of CNS. We observed high prevalence of methicillin resistance as well as the presence of both constitutive and inducible MLS_B_ phenotype in these clinical CNS isolates. However, the result showed no significant difference in the prevalence of antibiotic resistance between biofilm producers and non-producers CNS.

## Data Availability

Not applicable.

## Abbreviations

AST: Antibiotic susceptibility test
BA: Blood agar
BHI: Brain heart infusion
CLSI: Clinical and Laboratory Standards Institute
cMLS_B_: Constitutive macrolide, lincosamide, streptogramin B
CNS: Coagulase-negative staphylococci
CRA: Congo red agar
CVC: Central venous catheter
DJ stent: Double J stent
D-test: Constitutive and inducible clindamycin resistance test
iMLS_B_: Inducible macrolide, lincosamide, streptogramin B
MHA: Mueller Hinton agar
MLS_B_: Macrolide, lincosamide, streptogramin B
MRCNS: Methicillin resistant coagulase-negative staphylococci
MSA: Mannitol salt agar
MSCNS: Methicillin sensitive coagulase-negative staphylococci
MSCRAMMs: Microbial surface components recognizing adhesive matrix molecules
PBS: Phosphate buffer saline
PCR: Polymerase chain reaction
PIA: Polysaccharide intercellular adhesion
TCP: Tissue culture plate
TM: Tube adherence method
TSB: Tryptic soy broth
